# Chronic Hypoxia in Ovine Pregnancy Recapitulates Physiological and Molecular Markers of Preeclampsia in the Mother, Placenta, and Offspring

**DOI:** 10.1161/HYPERTENSIONAHA.122.19175

**Published:** 2022-05-09

**Authors:** Wen Tong, Beth J. Allison, Kirsty L. Brain, Olga V. Patey, Youguo Niu, Kimberley J. Botting, Sage G. Ford, Tessa A. Garrud, Peter F.B. Wooding, Caroline J. Shaw, Qiang Lyu, Lin Zhang, Jin Ma, Tereza Cindrova-Davies, Hong Wa Yung, Graham J. Burton, Dino A. Giussani

**Affiliations:** Department of Physiology Development & Neuroscience, University of Cambridge, United Kingdom (W.T., B.J.A., K.L.B., O.V.P., Y.N., K.J.B., S.G.F., T.A.G., P.F.B.W., T.C.-D., H.W.Y., G.J.B., D.A.G.).; Centre for Trophoblast Research, University of Cambridge, United Kingdom (W.T., Y.N., K.J.B., T.A.G., P.G.B.W., T.C.-D., H.W.Y., G.J.B., D.A.G.).; BHF Cardiovascular Centre for Research Excellence, University of Cambridge, United Kingdom (Y.N., K.J.B., D.A.S.).; Department of Aerospace Physiology, Fourth Military Medical University, Xi’an, China (Y.N., Q.L., L.Z., J.M., D.A.G.).; Department of Metabolism, Digestion and Reproduction, Imperial College London, United Kingdom (C.J.S.).

**Keywords:** endoplasmic reticulum, hypoxia, oxidative stress, placenta, unfolded protein response

## Abstract

Preeclampsia continues to be a prevalent pregnancy complication and underlying mechanisms remain controversial. A common feature of preeclampsia is utero-placenta hypoxia. In contrast to the impact of hypoxia on the placenta and fetus, comparatively little is known about the maternal physiology.

**Methods:**

We adopted an integrative approach to investigate the inter-relationship between chronic hypoxia during pregnancy with maternal, placental, and fetal outcomes, common in preeclampsia. We exploited a novel technique using isobaric hypoxic chambers and in vivo continuous cardiovascular recording technology for measurement of blood pressure in sheep and studied the placental stress in response to hypoxia at cellular and subcellular levels.

**Results:**

Chronic hypoxia in ovine pregnancy promoted fetal growth restriction (FGR) with evidence of fetal brain-sparing, increased placental hypoxia-mediated oxidative damage, and activated placental stress response pathways. These changes were linked with dilation of the placental endoplasmic reticulum (ER) cisternae and increased placental expression of the antiangiogenic factors sFlt-1 (soluble fms-like tyrosine kinase 1) and sEng (soluble endoglin), combined with a shift towards an angiogenic imbalance in the maternal circulation. Chronic hypoxia further led to an increase in uteroplacental vascular resistance and the fall in maternal blood pressure with advancing gestation measured in normoxic pregnancy did not occur in hypoxic pregnancy.

**Conclusions:**

Therefore, we show in an ovine model of sea-level adverse pregnancy that chronic hypoxia recapitulates physiological and molecular features of preeclampsia in the mother, placenta, and offspring.

**Novelty and Relevance**

**What Is New?**

A common feature of preeclampsia is placental hypoxia. However, whether placental hypoxia is merely a consequence of the disease or whether it causes the adverse maternal and fetal outcomes is uncertain.
Here, we show using a novel in vivo approach that placental hypoxia drives maternal and fetal phenotypes associated with preeclampsia.

**What Is Relevant?**

Pregnancy affected by uteroplacental hypoxia, one of the most common pregnancy complications leading to fetal growth restriction in humans, increases the risk of physiological and molecular markers of preeclampsia.

**Clinical and Pathophysiological Implications?**

Chronic hypoxia in ovine pregnancy recapitulates markers of preeclampsia in the mother, placenta, and offspring, indicating that placental hypoxia is an initiating factor in the pathoetiology of preeclampsia. Therefore, biomarkers of placental hypoxia, oxidative stress, and activation of the unfolded protein response must be addressed to guide future clinical management of preeclampsia.

Preeclampsia remains a leading cause of perinatal morbidity and mortality, affecting 2% to 8% of pregnancies worldwide.^1^ Therefore, there is ongoing interest in improving our understanding of the disease. Historically, preeclampsia was thought to develop exclusively in early pregnancy due to failure of spiral artery conversion and reduced uteroplacental perfusion.^2^ However, knowledge has expanded and it is now accepted that preeclampsia encompasses a broader spectrum of disorders, including early- and late-onset preeclampsia.^3^

Irrespective of cause, all forms of preeclampsia resolve after delivery of the placenta, confirming central involvement of the organ. In addition, most forms of preeclampsia present with evidence of impaired uteroplacental perfusion and placental hypoxia.^2^ However, whether placental hypoxia is merely a consequence of the disease or whether it causes the adverse maternal and fetal outcomes is uncertain. In contrast to the impact of hypoxia on the placenta and fetus, comparatively little is known about the maternal physiology. When investigating interactions among mother, placenta, and offspring, maternal metabolism, the temporal profile of fetal development, and access to longitudinal physiological measures are three important considerations. Sheep and humans share a similar precocial profile of organ development, and sheep give birth primarily to singleton or twin lambs of similar weight to humans after a relatively long gestation period.^4^ Therefore, the maternal and placental metabolic investment in pregnancy is similar between sheep and humans. In addition, sheep permit longitudinal assessment of uterine blood flow via surgically implanted flow probes as well as serial long-term blood sampling for endocrinology.^5^ In this study, we have tested the hypothesis that placental hypoxia drives phenotypes of preeclampsia by investigating the effects on mother, placenta, and fetus of chronic hypoxia during late pregnancy. To achieve this, we developed a preclinical model of improved human translational potential to investigate the symptoms of preeclampsia in late gestation in sheep, independent of maladaptive placental changes in early pregnancy.

## Materials and Methods

The authors declare that all supporting data are available within the article (and its Supplemental Material).

**Figure 1.**
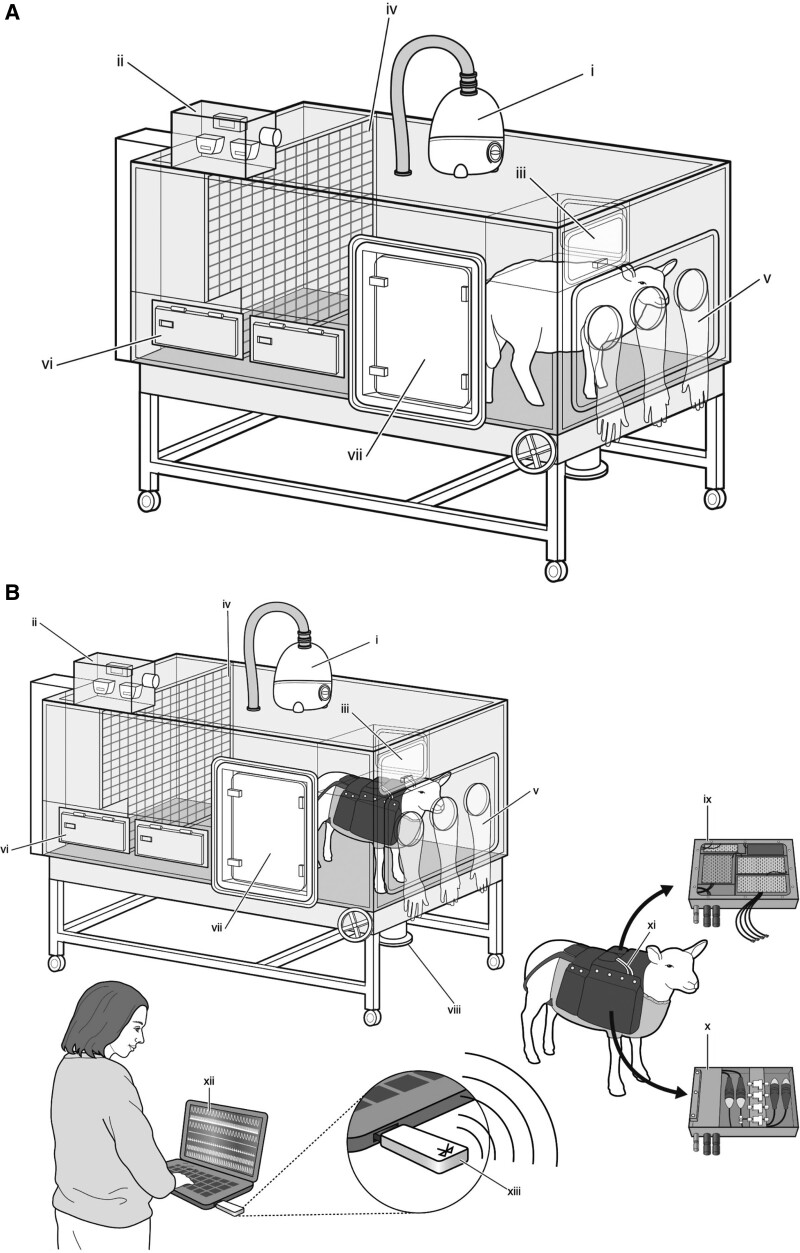
**Isobaric hypoxic chambers and wireless recording CamDAS system. A** and **B**, A specially designed nitrogen-generating system supplied compressed air and nitrogen to the bespoke isobaric hypoxic chambers housed at The Barcroft Centre, University of Cambridge. Each chamber was equipped with an electronic servo-controlled humidity cool steam injection system to return the appropriate humidity to the inspirate (i). Ambient partial pressures of oxygen and carbon dioxide, humidity, and temperature within each chamber were monitored via sensors (ii). For experimental procedures, each chamber had a double transfer port (iii) to internalize material and a manually operated sliding panel (iv) to bring the ewe into a position, where daily sampling of blood could be achieved through glove compartments (v). Each chamber incorporated a drinking bowl with continuous water supply and a rotating food compartment (vi) for determining food intake. A sealed transfer isolation cart could be attached to a side exit (vii) to couple chambers together for cleaning. Waste could be disposed via a sealable pipe (viii). **B**, A separate cohort of ewes was instrumented with the CamDAS system during surgery, allowing continuous longitudinal monitoring of arterial blood pressure and uterine blood flow. The wireless CamDAS system was contained in two parts in a custom-made sheep jacket: the data acquisition box (ix) on one side and a box containing the pressure transducers (x) on the other side. Cables (xi) provided connection between the two boxes and to 2 battery packs. Measurements made using the CamDAS system were transmitted wirelessly via Bluetooth technology (xiii) to a laptop on the outside (xii), on which it was possible to continuously measure and record uterine blood flow and maternal arterial blood pressure during the experimental period. Reproduced from Brain et al^6^ with permission. Copyright ©2015, John Wiley and Sons. Reproduced from Allison et al^7^ with permission. Copyright ©2016, John Wiley and Sons.

For the purpose of the current study, we exploited recently available novel technology to maintain pregnant sheep under highly controlled isobaric hypoxic conditions while undergoing wireless recording of maternal cardiovascular function (Figure 1).^6–8^ Then, we combined measurements in vivo with functional and molecular analyzes to determine the inter-relationship between chronic hypoxia and maternal, placental, and fetal outcomes. The experimental design was conducted in accordance with the ARRIVE (Animal Research: Reporting of In Vivo Experiments) guidelines.^9^

An expanded version of the Materials and Methods is available in the Supplemental Material.

## Results

### Chronic Hypoxia Causes Asymmetrical Fetal Growth Restriction

**Figure 2.**
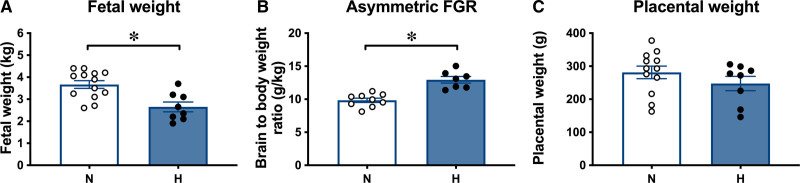
**Hypoxic pregnancy causes asymmetrical fetal growth restriction (FGR).** Values are mean±SEM for fetal weight (**A**), the ratio of fetal brain to body weight (**B**), and placental weight (**C**). Groups are normoxic (N; ○, n=9–13) and hypoxic (H; ●, n=7–8). Significant differences (*P*<0.05) are *N vs H, Student *t* test for unpaired data.

Exposure of pregnant ewes to chronic isobaric hypoxia of 10% inspired oxygen for a month from 105 to 138 days gestational age (dGA; term at 145 dGA) was associated with a 28% reduction in fetal growth, decreasing fetal weight from 3.67±0.17 kg in normoxic fetuses to 2.65±0.22 kg in hypoxic fetuses at 138 dGA (Figure 2A). There was no change in fetal brain weight in hypoxic relative to normoxic pregnancies (normoxic: 47.7±0.9 versus hypoxic: 47.8±1.3 g). However, when fetal brain weight was expressed relative to fetal body weight, this ratio was significantly increased in hypoxic relative to normoxic fetuses (Figure 2B). In contrast, there was no effect of chronic hypoxia on placental weight (Figure 2C) or on the number or weight distribution of different placentome types (Figure S1). These effects of chronic hypoxia on fetal growth occurred in the absence of changes to maternal food intake (Figure S2).

### Chronic Hypoxia Leads to Activation of the Placental Unfolded Protein Response

**Figure 3.**
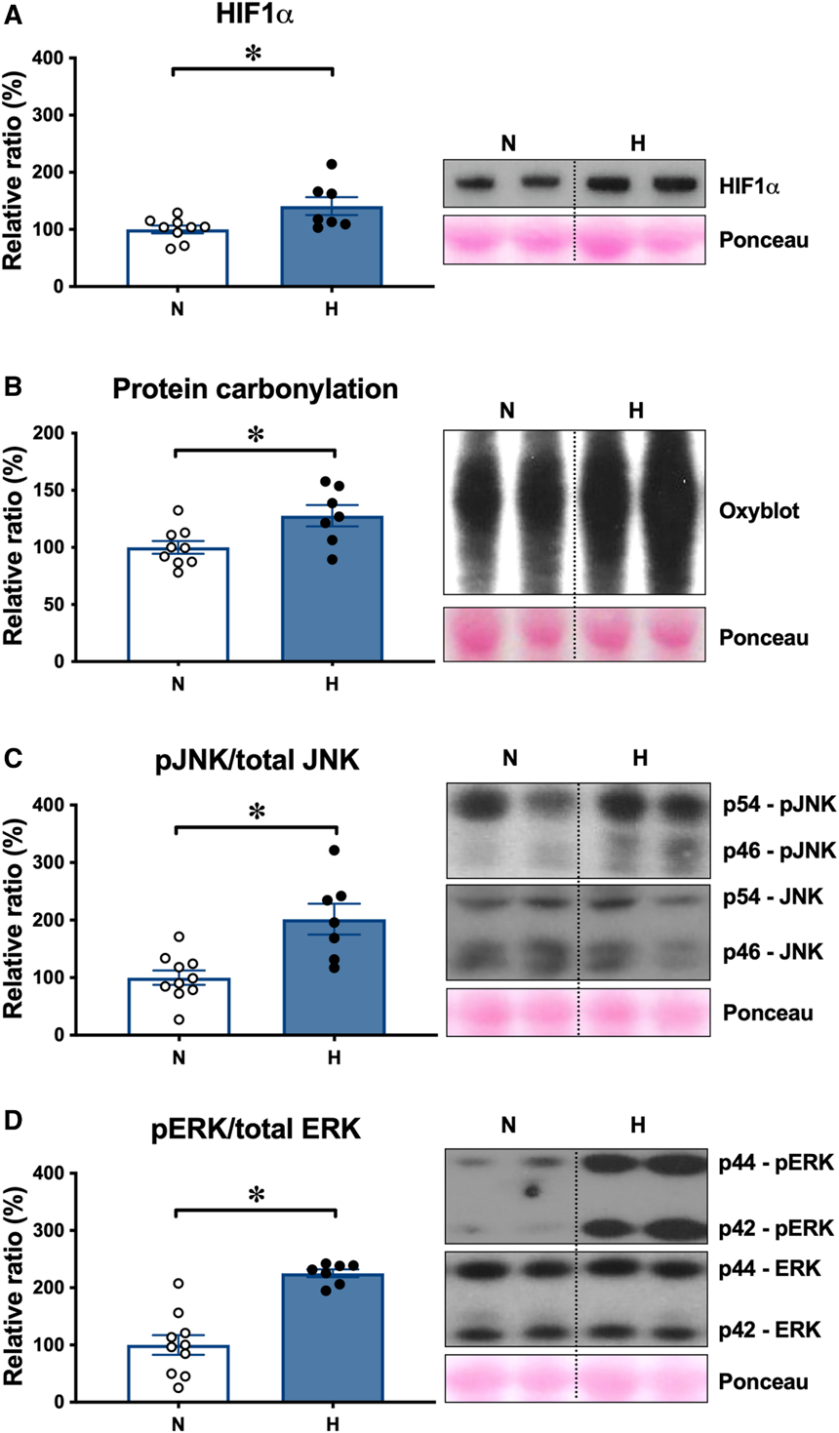
**Hypoxic pregnancy activates the placental stress response.** Values are mean±SEM for the relative ratio of placental levels of HIF1α (hypoxia-inducible factor 1α; **A**), of protein carbonylation (**B**), of the ratio of phosphorylated to total stress kinases JNK (C-jun normoxic-terminal kinase; **C**) and ERK (extracellular signal-regulated kinase; **D**). Blots for JNK and ERK appear atypical as they were resolved on 14% agarose gels for higher resolution. Groups are normoxic (N; ○, n=9–10) and hypoxic (H; ●, n=7). Significant differences (*P*<0.05) are *N vs H, Student *t* test for unpaired data. pERK indicates phosphorylated ERK; and pJNK, phosphorylated JNK.

**Figure 4.**
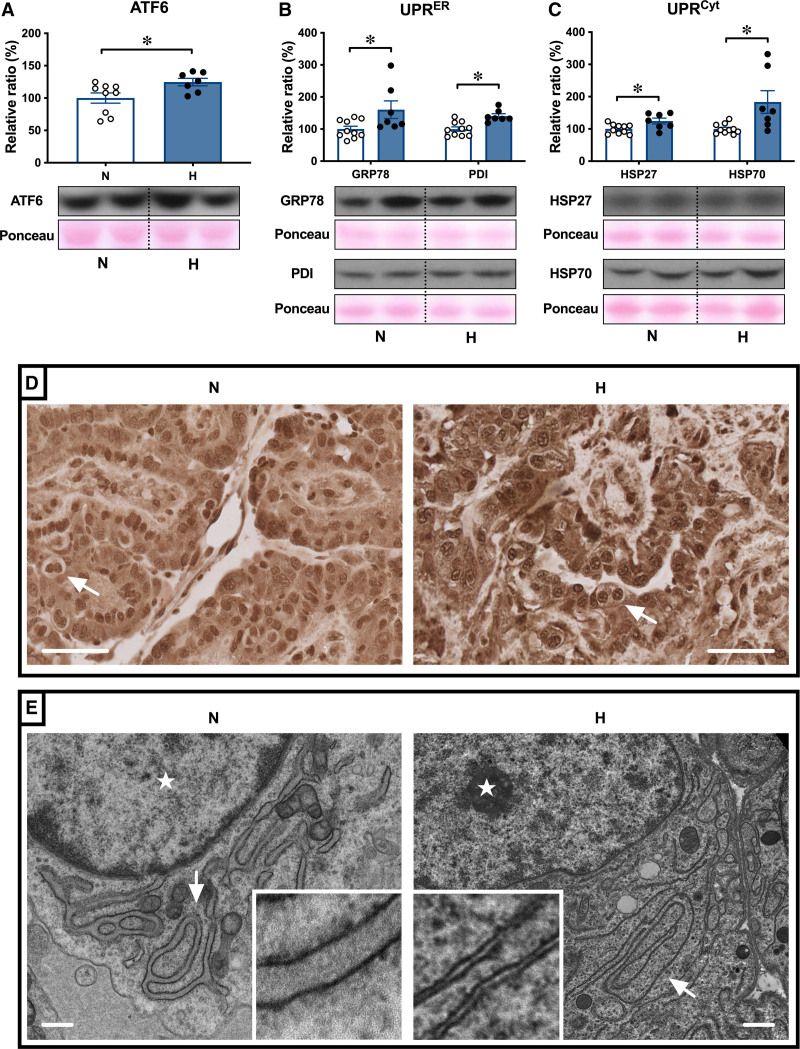
**Hypoxic pregnancy activates the placental UPR (unfolded protein response).** Values are mean±SEM for the relative ratio of the placental levels of ATF6 (activating transcription factor 6; **A**), GRP78 (glucose-related protein 78) and PDI (protein disulfide isomerase; **B**), and HSP27 (heat shock protein 27) and HSP70 (heat shock protein 70; **C**). Groups are normoxic (N; ○, n=9–10) and hypoxic (H; ●, n=7). Significant differences (*P*<0.05) are *N vs H, Student *t* test for unpaired data. In the placenta, ATF6 localizes to the nuclei (**D**), with more prominent nuclear staining in H compared with N placentae. Pictured (**D**) trophoblast containing binucleate cells (arrows); scale bar=50 µm. Change in trophoblast endoplasmic reticulum (ER) structure was examined by transmission electron microscopy (**E**). Representative images taken at ×5000 magnification are shown. Arrows indicate the location of ER and stars indicate the location of the nucleus; scale bar=500 nm.

The levels of HIF1α (hypoxia-inducible factor 1α) were greater in hypoxic relative to normoxic placentomes at 138 dGA (Figure 3A). The levels of protein carbonylation were greater in hypoxic relative to normoxic placentae (Figure 3B), indicative of oxidative stress. This was associated with an increase in the ratio of the phosphorylated forms of the mitogen-activated protein kinases JNK (C-jun normoxic-terminal kinase) and the cell survival ERK (extracellular signal-regulated kinase) compared with total levels of these kinases in hypoxic relative to normoxic placentae (Figure 3C and 3D). Oxidative protein damage can trigger activation of UPR (unfolded protein response) pathways in different cellular compartments, including the cytosol and endoplasmic reticulum (ER). The UPR signal activator ATF6 (activating transcription factor 6) was increased in hypoxic relative to normoxic placentae (Figure 4A). As part of the ER UPR, levels of the protein chaperone GRP78 (glucose-related protein 78) and of the protein folding enzyme PDI (protein disulfide isomerase) were higher in hypoxic relative to normoxic placentae (Figure 4B). The expression of the cytosolic protein chaperones HSP27 (heat shock protein 27) and HSP70 (heat shock protein 70), part of the cytosolic UPR, was also greater in hypoxic relative to normoxic placentae (Figure 4C). Immunohistochemical analysis showed that ATF6 localized to the nucleus, indicating potential transcriptional activity of ATF6 (Figure 4D). Nuclear staining was more prominent in hypoxic compared with normoxic placentae (Figure 4D). Transmission electron microscopy further revealed distended ER morphology in hypoxic compared with the ER in normoxic placentae, which displayed a highly defined membrane structure (Figure 4E).

### Chronic Hypoxia Promotes an Angiogenic Imbalance in the Maternal Circulation

**Figure 5.**
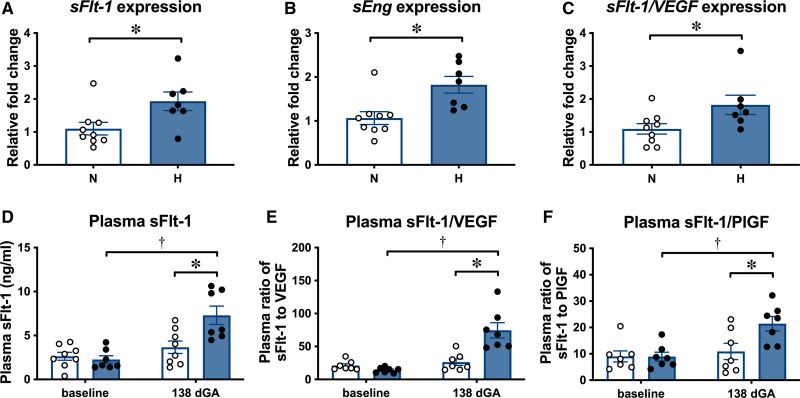
**Hypoxic pregnancy changes placental expression of antiangiogenic factors, increasing the antiangiogenic to proangiogenic balance in maternal plasma. Upper**, Values are mean±SEM for the relative placental fold change for sFlt-1 (soluble fms-like tyrosine kinase 1; **A**), sEng (soluble endoglin; **B**), and the ratio of sFlt-1 to VEGF (vascular endothelial growth factor; **C**). **Lower**, Values are mean±SEM for plasma concentration of sFlt-1 (**D**) and plasma ratios of sFlt-1 to PlGF (placental growth factor; **E**) and to VEGF (**F**). Groups are normoxic (N; ○, n=7–9) and H (●, n=7). Significant differences (*P*<0.05) are *N vs H or † vs baseline; Student *t* test for unpaired data or 2-way repeated-measures ANOVA where appropriate. dGA indicates days gestational age.

Placental transcripts encoding the antiangiogenic factors sFlt-1 (soluble fms-like tyrosine kinase 1) and sEng (soluble endoglin), as well as the ratio of the placental transcripts of sFlt-1 compared with the angiogenic factor VEGF (vascular endothelial growth factor), were increased in hypoxic relative to normoxic placentae at 138 dGA, as measured by quantitative reverse transcription polymerase chain reaction (Figure 5A through 5C). There were no differences in *VEGF* and PlGF (placental growth factor) transcripts, and no differences in the ratio of *sFlt-1* compared with *PlGF* transcripts (Figure S3A through S3C). In normoxic ewes, the concentration of sFlt-1 and the ratios of sFlt-1 to VEGF and to PlGF in plasma did not change in samples taken at baseline and at 138 dGA (Figure 5D through 5F). In contrast, in hypoxic ewes, the concentrations of sFlt-1 and the ratios of sFlt-1 to VEGF and to PlGF were significantly higher at 138 dGA relative to baseline and when compared with values in normoxic ewes at 138 dGA (Figure 5D through 5F). Neither normoxic nor hypoxic ewes showed changes in sEng, VEGF, or PlGF plasma concentrations with increasing gestation, and there were no differences between the groups at baseline or 138 dGA (Figure S3D through S3F).

### Chronic Hypoxia Increases Uteroplacental Vascular Resistance and Prevents the Gestational Decrease in Maternal Arterial Blood Pressure

**Figure 6.**
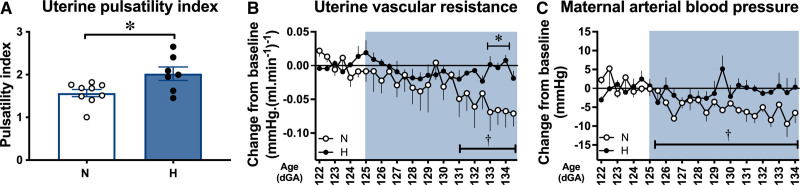
**Hypoxic pregnancy causes maternal cardiovascular dysfunction.** Values are mean±SEM for uterine artery pulsatility index (PI) (**A**) and the change from baseline in uterine vascular resistance (**B**) and arterial blood pressure (**C**). Groups are normoxic (N; ○, n=5–9), hypoxic (H; ●, n=5–7). Significant differences (*P*<0.05) are *N vs H, or † vs baseline; Student *t* test for unpaired data or 2-way repeated-measures ANOVA, where appropriate. dGA indicates days gestational age.

At 138 dGA, the uterine artery pulsatility index (PI) values were greater in hypoxic relative to normoxic ewes (Figure 6A). At 138 dGA, independent of treatment, there were significant positive correlations between maternal uterine PI and the maternal plasma sFlt-1 concentration, and between maternal uterine PI and maternal plasma sFlt-1 to PlGF ratio (Figure S4A and S4C). However, there was no correlation between maternal uterine PI and maternal plasma sFlt-1 to VEGF ratio at 138 dGA (Figure S4B). At 138 dGA, plasma creatinine concentrations were slightly higher in hypoxic relative to normoxic ewes, which may indicate a reduction in glomerular filtration rate (Figure S5A). However, there was no difference in the urine ratio of albumin to creatinine at 138 dGA (Figure S5B).

Daily changes in maternal arterial blood gas, acid base, and metabolic status during exposure to chronic normoxia or chronic hypoxia in the second cohort of animals have been previously reported.^7^ In brief, these data confirm a reduction in maternal arterial partial pressure of oxygen from 105.7±3.7 to 42.0±1.2 mm Hg and in arterial oxygen saturation 103.5±0.5 to 78.6±5.7% (*P*<0.05) during exposure to chronic isobaric hypoxia of 10% inspired oxygen (Table S1). Ewes exposed to chronic hypoxia had significantly elevated hematocrit and hemoglobin concentration by the end of exposure relative to baseline and to values in normoxic ewes (Table S1). There was no significant change between groups in maternal arterial pH, partial pressure of arterial carbon dioxide, blood glucose, or lactate concentrations (Table S1).

Longitudinal continuous measurement of maternal arterial blood pressure and calculation of uterine vascular resistance via the wireless CamDAS recording revealed the expected fall in both variables with advancing gestation in normoxic ewes but not in hypoxic ewes (Figure 6B and 6C). During baseline conditions, before hypoxic exposure, between 122 and 124 dGA, average values for maternal arterial blood pressure (80.4±1.2 versus 81.2±3.2 mm Hg) and for uterine vascular resistance (0.29±0.05 versus 0.20±0.03 mm Hg·[mL·min^-1^]^-1^) were not different between normoxic and hypoxic ewes. In normoxic pregnancies, values for maternal arterial blood pressure (80.4±1.2 versus 73.4±1.1 mm Hg) and for uterine vascular resistance (0.29±0.05 versus 0.20±0.06 mm Hg·[mL·min^-1^]^-1^) were significantly lower at 134 dGA compared with earlier in gestation (all *P*<0.05). In contrast, following chronic hypoxic exposure, values for maternal arterial blood pressure (81.2±3.2 versus 79.6±4.8 mm Hg) and for uterine vascular resistance (0.20±0.03 versus 0.19±0.04 mm Hg·(mL·min^-1^)^-1^) in hypoxic pregnancies were similar at 134 dGA compared with earlier in gestation (*P*>0.05). Maternal arterial blood pressure was not significantly different between normoxic and hypoxic pregnancies at 134 dGA.

## Discussion

In the classic 2-stage theory of preeclampsia, impaired uteroplacental perfusion promotes placental hypoxia, oxidative stress, and ER stress. The subsequent placental release of proinflammatory cytokines, syncytiotrophoblast debris, and antiangiogenic factors into the maternal circulation induces the peripheral syndrome.^10^ For example, the angiogenic imbalance caused by the release of sFlt-1 and sEng, which oppose the actions of VEGF and PlGF, promotes global maternal endothelial dysfunction and increased peripheral vascular resistance.^11^ These changes manifest as an increase in the uterine PI, maternal systemic hypertension, and impaired renal glomerular filtration rate.^12^ On the fetal side, these changes lead to impaired fetal oxygen and nutrient delivery, yielding asymmetrical fetal growth restriction (FGR).^13^

Data in the present study show that chronic hypoxia during the last third of pregnancy in sheep stresses the placenta, with upstream adverse effects on the mother and downstream adverse effects on the fetus, akin to those found in preeclampsia. Placentae from the hypoxic cohort showed molecular evidence of hypoxia, increased oxidative stress, activation of the UPR, dilation of ER cisternae, and increased expression of antiangiogenic factors. Upstream adverse consequences on the ewe included evidence of an angiogenic imbalance in maternal plasma, increased uterine artery PI, and a lack of an ontogenic fall in uterine vascular resistance and arterial blood pressure with advancing gestation. Downstream adverse consequences on the hypoxic offspring included FGR with evidence of fetal brain-sparing. Combined, therefore, the data in this study support the hypothesis that chronic hypoxia during the last third of pregnancy in sheep provides a link between placental stress, FGR, and maternal cardiovascular dysfunction in adverse pregnancy, as in preeclampsia. However, the differences between this preclinical model and preeclampsia are just as informative as the similarities. Although the data suggest that some features of preeclampsia can be caused by hypoxia, other features, such as overt maternal hypertension and maternal proteinuria, were not recapitulated. However, ewes undergoing hypoxic pregnancy did not show the significant fall in maternal arterial blood pressure measured in control ewes with advancing gestation. Lack of maternal hypertension may, therefore, be due to the limited duration of hypoxia towards the end of pregnancy in this ovine model. In contrast, in preeclampsia, the pathophysiology can start during the first trimester.

### Chronic Hypoxia and Asymmetrical Fetal Growth Restriction

The level of maternal hypoxia used in this model is clinically relevant. Previous studies from our group have used the hypoxic chambers with pregnant sheep, which were surgically prepared with catheters for daily blood sampling and Transonic flow probes for long-term recording of fetal cardiovascular function.^7^ These studies revealed that the level of maternal hypoxia used in the present study reduced fetal arterial partial pressure of oxygen in the descending aorta to 12 mm Hg in a highly controlled manner.^7^ This level of chronic hypoxia equates to that measured by cordocentesis in human growth-restricted fetuses in preeclamptic pregnancies.^14^ Our previous studies also revealed that chronic fetal hypoxia promotes a sustained redistribution of blood flow away from the peripheral circulations towards the fetal brain.^7,15^ This is the so-called fetal brain-sparing effect^16^ and is responsible for the asymmetrical FGR measured in chronically hypoxic fetuses both in humans and animal models.^15,17^ In the present study, the asymmetrical FGR resulting from chronic hypoxia during the last third of pregnancy was represented by a smaller fall in the brain relative to the fetal body weight, yielding an increase in the percentage relative brain weight.

### Chronic Hypoxia and Placental Stress

Placental hypoxia promotes an increase in placental oxidative stress.^18^ Protein carbonyls are used as biomarkers of reactive oxygen species–mediated protein damage in preeclamptic placentae and correlate well with the severity of the syndrome.^19^ Accumulation of damaged proteins in the placenta is associated with activation of the kinases ERK and JNK, which mediate several responses to cellular stress.^20^ As part of the cellular quality control system, the ER ensures protein folding and is capable of activating a powerful UPR to restore protein homeostasis.^21^ In the present study, both the ER UPR and cytosolic UPR showed increased activation in hypoxic placentae, along with morphological changes in ER structure. We found that both the expression and nuclear translocation of ATF6 was increased, likely mediating the transcriptional activation of UPR target genes in response to ER stress.^22^ In hypoxic placentae, the expression of GRP78 and PDI and of HSP27 and HSP70 were increased as part of the ER UPR and the cytosolic UPR, respectively. Transmission electron microscopy further revealed distended ER cristae in hypoxic placentae. Many of these molecular and morphological markers have been reported in placentae from women with preeclampsia.^23–25^

### Chronic Hypoxia and Maternal Adverse Effects

Healthy human pregnancy is accompanied by several maternal cardiovascular adaptations that help support the growing fetus.^26^ By midgestation, there is a fall in uteroplacental vascular resistance, which directs perfusion towards the uterine artery, where blood flow is increased from 20 to 50 mL/min in the nonpregnant state to 450 to 800 mL/min.^26^ To accommodate this, the uterine artery markedly increases its diameter, driving a fall in uterine vascular resistance and maternal arterial pressure.^27^ Pregnancy at high-altitude blunts the rise in uterine blood flow and impairs the fall in maternal arterial blood pressure with advancing gestation in nonindigenous human populations^28,29^ and sheep.^30,31^ The diminished rise in uterine blood flow in human highland pregnancy is thought to be an important contributor to the enhanced prevalence of preeclampsia and FGR at high altitude.^32,33^ Extensive studies by Hu et al^30,34^ have shown that gestational hypoxia contributes to the maladaptive uterine hemodynamic phenotype through epigenetic regulation of the large-conductance calcium-activated potassium channel.

Data in the present study show that chronic hypoxia during the last third of pregnancy in sheep led to an increase in placental sFlt-1 expression and maternal plasma sFlt-1 concentration. This may be driven by increased placental levels of HIF1α in the hypoxic placenta, which has been previously demonstrated in vitro in placental explants and is supported by raised HIF1α levels in the current study.^35^ In addition, the fall in uterine vascular resistance and maternal blood pressure with advancing gestation monitored using indwelling flow probes and vascular catheters did not occur in hypoxic ewes. Both uterine vascular dysfunction and increased maternal blood pressure have been reported in sheep undergoing high-altitude pregnancy.^34^ Therefore, combined, the present study extends previous findings in ovine highland pregnancy,^34^ highlighting the critical role of oxygen deficiency in placental dysfunction and their relationship with maternal cardiovascular changes. Furthermore, there was a significant positive correlation between maternal uterine PI and the maternal plasma concentration of sFlt-1 and between maternal uterine PI and the maternal plasma sFlt-1 to PlGF ratio. The data, therefore, support that increased expression of antiangiogenic factors in the placenta may contribute to an angiogenic imbalance and endothelial dysfunction in the maternal circulation.

### Advances and Limitations

Despite great advances in the understanding of preeclampsia, progress in this field has been hampered by many experimental limitations. Although advances, such as organoid cultures create new and exciting opportunities, in vitro models cannot replicate all in vivo interactions between mother, placenta, and offspring. However, there are no preclinical animal models that spontaneously develop preeclampsia, and those available, in which symptoms are induced, have limitations. The same is true for this ovine model of hypoxic pregnancy. Clearly, there are gross anatomic differences between the human hemochorial and the ovine synepitheliochorial placenta.^36^ However, there are also important similarities. Both sheep and humans have placental counter-current flow of maternal and fetal blood within the placental villous tree, comparable transplacental oxygen gradients and oxygen consumption rates (37 in sheep versus 34 mL·kg^-1^·min^-1^ in humans), as well as similar nutrient transporter expression.^37–41^ At the molecular level, the induction of oxidative and ER stress and the activation of the UPR are highly conserved pathways across species.^21^ This is also the case for rodents, in which activation of the placental ER UPR has been demonstrated in hypoxic pregnancy.^34^

There have been many other studies by our and other groups in rodent pregnancy investigating the effects of hypoxic pregnancy on the placental phenotype, FGR, and uterine vascular reactivity.^42–46^ However, few of these studies have had a focus on placental molecular or maternal circulating markers of preeclampsia or investigated associated changes in maternal in vivo cardiovascular function. It is also important to highlight that the murine placenta is functionally divided into distinct zones for endocrine activity and nutrient transfer.^47^ The labyrinth zone shows high levels of mitochondrial activity, while the junctional zone is less well oxygenated, but prone to ER stress due to its synthetic and secretory activities.^48^ Thus, in the murine placenta, crosstalk between ER and mitochondrial stress is limited, with diverging responses depending on which zone is investigated.^49,50^ Other murine studies using experimental models of preeclampsia by either endothelial NO synthase knockout or restriction of uteroplacental perfusion also support a link between placental hypoxia with oxidative stress and impaired placental nutrient transport, FGR, and abnormal maternal cardiovascular function.^49,51^ This is what we have directly addressed in the present study in an ovine model with increased human translational potential. Nevertheless, extrapolation of these findings to the human clinical condition needs to be viewed with caution.

### Perspectives

This work introduces a novel large animal model of isobaric hypoxic pregnancy in sheep that not only promotes FGR but also recapitulates many of the physiological and molecular features of preeclampsia in the mother and the placenta. These findings are significant, as any changes occur independently of alterations to placentation in early pregnancy. Therefore, the work offers novel ways of thinking about the syndrome and an established platform to develop interventional therapies.

## Article Information

### Acknowledgments

W. Tong is a PhD student supported by the Centre for Trophoblast Research at the University of Cambridge. We are grateful to the staff of the University of Cambridge Biological Services for helping with the maintenance of the animals at The Barcroft Centre.

### Article Contributions

D.A. Giussani contributed to conceptualization. W. Tong, B.J. Allison, K.L. Brain, O.V. Patey, Y. Niu, K.J. Botting, S.G. Ford, T.A. Garrud, F.B.P. Wooding, Q. Lyu, L. Zhang, J. Ma, T. Cindrova-Davies, H. Wa Yung, G.J. Burton, and D.A. Giussani contributed to Methodology. W. Tong, B.J. Allison, K.L. Brain, O.V. Patey, Y. Niu, K.J. Botting, S.G. Ford, T.A. Garrud, F.B.P. Wooding, Q. Lyu, L. Zhang, J. Ma, T. Cindrova-Davies, H. Wa Yung, G.J. Burton, and D.A. Giussani participated in formal analysis. W. Tong, B.J. Allison, K.L. Brain, and D.A. Giussani contributed to writing the original draft. W. Tong, B.J. Allison, K.L. Brain, O.V. Patey, Y. Niu, K.J. Botting, S.G. Ford, T.A. Garrud, F.B.P. Wooding, Q. Lyu, L. Zhang, J. Ma, T. Cindrova-Davies, H. Wa Yung, and G.J. Burton contributed to writing—review and editing. W. Tong and D.A. Giussani contributed to visualization. W. Tong, B.J. Allison, K.L. Brain, O.V. Patey, Y. Niu, K.J. Botting, S.G. Ford, T.A. Garrud, F.B.P. Wooding, Q. Lyu, L. Zhang, J. Ma, T. Cindrova-Davies, H. Wa Yung, G.J. Burton, and D.A. Giussani participated in supervision. W. Tong, B.J. Allison, K.L. Brain, O.V. Patey, Y. Niu, K.J. Botting, S.G. Ford, and D.A. Giussani contributed to project administration. D.A. Giussani and G.J. Burton participated in funding acquisition.

### Sources of Funding

This work was supported by The British Heart Foundation (RG/17/8/32924).

### Disclosures

None.

## Supplementary Material


