# Supplementary material for: Chronic Hypoxia in Ovine Pregnancy Recapitulates Physiological and Molecular Markers of Preeclampsia in the Mother, Placenta, and Offspring
Source: Hypertension. 2022 May 9;79(7):1525–35. doi: 10.1161/HYPERTENSIONAHA.122.19175 (PMC9172902; doi:10.1161/HYPERTENSIONAHA.122.19175)
Supplement: Supplementary file 1 [file hyp-79-1525-s001.pdf]

Chronic hypoxia in ovine pregnancy recapitulates physiological and molecular markers of preeclampsia in the mother, placenta and offspring

Wen Tong[1,2], Beth J. Allison[1], Kirsty L. Brain[1], Olga V. Patey[1], Youguo Niu[1-5], Kimberley J. Botting[1-4], Sage G. Ford[1], Tess A. Garrud[1,2], Peter F. B. Wooding[1,2], Caroline J. Shaw[6], Qiang Lyu[5], Lin Zhang[5], Jin Ma[5], Tereza Cindrova-Davies[1-3], Hong Wa Yung[1-3], Graham J. Burton[1-3], Dino A. Giussani*[1-5]

[1]*Department of Physiology Development & Neuroscience, University of Cambridge, United Kingdom;*

[2]*Centre for Trophoblast Research, University of Cambridge, United Kingdom*;

[3]*Strategic Research Initiative in Reproduction, University of Cambridge, United Kingdom*

[4]*BHF Cardiovascular Centre for Research Excellence, University of Cambridge, United Kingdom*

[5]*Department of Aerospace Physiology, Fourth Military Medical University, Xi'an, China;*

[6]*Department of Metabolism, Digestion and Reproduction, Imperial College London, United Kingdom*

Short Title: Pre-eclampsia link to gestational hypoxia

Corresponding Author: Professor Dino A. Giussani PhD ScD FRCOG
Department of Physiology, Development and Neuroscience
University of Cambridge
Downing Street, Cambridge
CB2 3EG, UK
Email: dag26@cam.ac.uk
Tel: +44 (0)1223 333894

Online supplement

Expanded Materials & Methods

Ethical approval

All procedures were performed at The Barcroft Centre of The University of Cambridge under the UK Animals Scientific Procedures Act 1986 and were approved by the Ethical Review Board of the University of Cambridge. The experimental design was conducted in accordance with the ARRIVE guidelines [1].

Exposure to chronic hypoxia

Pregnant Welsh mountain ewes carrying singleton fetuses determined by ultrasound scan at 80 days gestational age (dGA; Toshiba Medical Systems Europe, Zoetermeer, Netherlands; term is *ca.* 145 days) were randomly assigned at 103 dGA to either chronic normoxia (N) or chronic hypoxia (H). From 103 dGA, N and H ewes were fed daily a bespoke maintenance diet consisting of concentrate and hay pellets, thereby facilitating the monitoring of food intake (Cambridge ewe diet: 40g nuts/kg and 3g hay/kg; Manor Farm Feeds Ltd; Oakham, Leicestershire, UK). At 103 dGA, H ewes were moved into one of four bespoke isobaric hypoxic chambers (Telstar Ace, Dewsbury, West Yorkshire, UK; Figure 1A in main text) housed in a laboratory. Ewes assigned to chronic normoxia were housed in individual floor pens with the same floor area as the hypoxic chambers. The hypoxic chambers were supplied with controlled volumes of nitrogen and air via a bespoke air and nitrogen generating system (Domnick Hunter Gas Generation, Gateshead, Tyne & Wear, UK), as previously described in detail [2-4]. In brief, compressed air and compressed nitrogen were piped to the laboratory and gases were mixed to requirements via flow metres prior to entering the chambers. The inspirate was passed via silencers able to reduce noise to levels below regulation, providing a tranquil environment for the animal inside each chamber. The volume of gas in each chamber underwent a minimum of 12 changes per hour. All chambers were equipped with humidifiers (1100-03239 HS-SINF Masalles, Barcelona, Spain) and ambient PO_2, PCO_2, humidity and temperature within each chamber were monitored via sensors and values recorded continuously via the Trends Building Management System of the University of Cambridge. In this way, the level of oxygen within each chamber could be controlled with precision longitudinally over long periods of time. At 105 dGA, H ewes were gradually subjected to hypoxia, reaching 10 ± 1% inspired oxygen over 24h. This level of hypoxia was maintained for a month until 138dGA. For blood sampling procedures during this period, materials could be introduced into the chambers via a double transfer port. A sliding panel was then manually operated, encouraging the ewe to the front of the chamber, which permitted blood samples to be taken via glove compartments using sterile techniques and without losing the hypoxic exposure (Figure 1). In both N and H ewes, venous samples (5 ml) were taken from the jugular vein at 103 and 105 dGA (baseline) and at 138 dGA (end of chronic exposure). Samples were centrifuged, snap frozen in liquid nitrogen and stored at -80 ºC for subsequent analysis.

Doppler ultrasonography

At 138 dGA, following blood sampling, N and H ewes were moved to a nearby ultrasound room. Ultrasonography was performed using a Toshiba Powervision 7000

System with a convex 3.75 MHz Toshiba PVK-357AT transducer. Colour Doppler was used to identify the uterine artery, and the uterine artery PI was calculated using colour Doppler flowmetry. An average value of three consecutive waveforms on both uterine arteries was used for analysis. Ewes in the H group were transported to the ultrasound room and underwent all ultrasound procedures maintaining the hypoxic exposure of 10 ± 1% inspired oxygen via a customised respiratory hood in a mobile cart unit. The gas mixture supplying the respiratory hood was the same ratio as the gas mixture of air and nitrogen supplying the chambers, adjusted to volume.

Post-mortem, tissue and urine collection

At 138 dGA, following ultrasonography, N and H ewes were moved to the post-mortem laboratory. Ewes in the H group remained hypoxic at 10 ± 1% inspired oxygen via the respiratory hood until euthanasia. Both N and H ewes were killed humanely by an overdose of sodium pentobarbitone (0.4 ml/kg intravenously, Pentoject; Animal Ltd., UK). A urine sample (5 ml) was taken with a syringe and needle via puncture directly from the bladder. The fetus was delivered by hysterotomy, weighed and measured. One group of scientists isolated the maternal and fetal organs, which were weighed and fixed in 4% paraformaldehyde or snap frozen in liquid nitrogen and stored at -80°C until further analysis.

Following hysterectomy, individual placentomes were isolated, weighed and counted. Typed placentomes were counted and weighed, and placentomes of each type were also fixed or frozen for further analysis. The distribution of different types of placentomes was not different between N and H pregnancies (Figure S1). For consistency, all placental analysis was performed on type A placentomes.

Placental studies

Measurement of protein carbonylation. Flash frozen A-type placentomes were homogenised to powder using pestle and mortar while keeping tissues frozen on dry ice. Homogenates and ice-cold cell lysis buffer (1 mL of buffer per 100 mg of tissue; Cell Signaling Technology, UK) containing protease inhibitors (Roche Diagnostics, UK) were used to prepare protein lysates. Protein concentration was determined using a bicinchoninic acid assay (Thermo Fisher Scientific, UK). To determine post-translational protein carbonylation as a result of oxidative damage, an OxyBlot™ analysis was performed, according to the manufacturer's instructions (Millipore, Billerica, MA). In short, protein lysates were treated to derivatise carbonyl groups to 2,4-dinitrophenyl-hydrazone moieties, separated by 12% sodium dodecyl sulfate polyacrylamide gel electrophoresis and then transferred onto a nitrocellulose membrane (Hybond® ECL™, Sigma-Aldrich, UK). Non-specific binding was inhibited by blocking the membrane in 2.5% bovine serum albumin in Tris-buffered saline containing 0.1% Tween 20 (TBS-T) for 1 h at room temperature. The nitrocellulose membrane was incubated with a primary rabbit anti-2,4-dinitrophenyl-hydrazone antibody (Chemicon Oxyblot™; diluted 1:200) for 1 h at room temperature. Following incubation with the primary antibody, membranes were washed with TBS-T and incubated with a secondary antibody conjugated with horseradish peroxidase against Rabbit IgG (Thermo Fisher, UK; diluted 1:10000) for 1 h at room temperature. Following further washing with TBS-T, protein levels were visualised using an enhanced chemiluminescence kit (Pierce™ ECL, Thermo Fisher Scientific, UK). Protein band densities were quantified using ImageJ software (NIH, RRID:SCR_003070) and normalised against Ponceau S staining.

Western blotting. To determine placental protein expression, whole cell lysates were prepared from homogenates of flash frozen A-type placentomes. This was performed using ice-cold cell lysis buffer (1 mL of buffer per 100 mg of tissue; Cell Signaling Technology, UK) containing protease inhibitors (Roche Diagnostics, UK). Protein concentration was determined using a bicinchoninic acid assay (Thermo Fisher Scientific, UK). Samples were mixed with sodium dodecyl sulfate gel loading buffer and denatured for five minutes at 70 ºC. 15-30 µg aliquots of protein were resolved on 10-12% sodium dodecyl sulfate polyacrylamide gel electrophoresis agarose gels, transferred onto nitrocellulose membranes (Hybond® ECL™, Sigma-Aldrich, UK) and stained with 0.1% Ponceau S in 5% acetic acid (Sigma-Aldrich, UK). Non-specific binding was inhibited by blocking the membrane in 5% dry skim milk in TBS-T for 1 h at room temperature. Following incubation with the primary antibody, membranes were washed with TBS-T and incubated with the relevant secondary antibodies conjugated with horseradish peroxidase against Rabbit IgG (Thermo Fisher, UK; diluted 1:10,000) or against Mouse IgG (Thermo Fisher, UK; diluted 1:10,000) for 1 h at room temperature, where appropriate. Following further washing with TBS-T, protein levels were visualised using an enhanced chemiluminescence kit (Pierce™ ECL, Thermo Fisher Scientific, UK) on film (Amersham™ Hyperfilm™ ECL, GE Healthcare, UK). Protein band densities were quantified using ImageJ software (NIH; RRID:SCR_003070) and normalised against Ponceau S staining. A full list of primary antibodies, dilutions and incubation times can be found in the Major Resources Table.

Immunohistochemistry**.** Formalin-fixed paraffin-embedded A-type placentomes were sectioned to 7 µm thickness using a microtome (Leica Biosystems, UK) and mounted onto Superfrost™ Plus microscope slides and incubated at 37 ºC overnight. Sections were rehydrated in tap water for 10 minutes and incubated in 3% hydrogen peroxide (Fisher Scientific) for 15 minutes to block endogenous peroxidase activity. After rinsing in distilled water, the sections were incubated in Tris-buffered saline containing 0.1% Tween 20 and 0.1% Triton X-100 (TBS-TT) for 30 minutes. After rinsing in Tris-buffered saline (TBS), slides were blocked in 5% bovine serum albumin in TBS for 1 hour and then incubated overnight in primary antibody against ATF6 (Abcam, UK; ab37149; RRID:AB 725571; diluted 1:200) in 5% bovine serum albumin. The following day, sections were washed using TBS-TT and then incubated in biotinylated secondary antibody against Rabbit IgG (Vector Laboratories, Canada; diluted 1:200) in 5% bovine serum albumin. After washing in TBS-TT, staining was visualised using the VECTASTAIN avidin-biotin complex method (Vector Laboratories, Canada) by adding metal DAB (Thermo Fisher Scientific, UK) as the chromogen for 5 minutes. Staining was stopped by rinsing in distilled water. Sections were dehydrated, and cover slips mounted using DPX Mountant (Sigma-Aldrich, UK).

Transmission Electron Microscopy. Tissue processing, embedding and sectioning was performed by the Cambridge Advanced Imaging Centre. In brief, small pieces of A-type placentome tissue were fixed by immersion in 2 mM calcium chloride in 0.05M Sodium cacodylate buffer at pH 7.4 containing 2% glutaraldehyde and 2% formaldehyde. The tissues were fixed overnight at 4°C and osmicated in 0.05M Sodium cacodylate buffer at pH 7.4 containing 1% osmium tetroxide and 1.5% potassium ferricyanide for three days at 4°C. They were treated with 0.1% thiocarbohydrazide for 20 min in the dark at room temperature. They were then osmicated a second time in 2% osmium tetroxide and stained in 0.05 maleate buffer

at pH 5.5 containing 2% uranyl acetate for three days at 4°C. The tissue was dehydrated in ascending concentrations to 100% ethanol and then in 100% dry acetone and dry acetonitrile. They were embedded in Quetol epoxy resin over the course of 11 days. 80 nm sections were cut on a Ultracut UCT (Leica, Germany) and mounted onto 400 mesh bare copper grids. Transmission electron microscopy was performed on a FEI Tecnai G2 transmission electron microscope run at 200 keV accelerating voltage and using a 20 µm objective aperture to improve contrast. Images were taken using an AMT camera at 5000-fold magnification.

Quantification of mRNA transcripts using quantitative RT-PCR. RNA was extracted from flash frozen A-type placentomes using QIAzol Lysis Reagent Solution and Qiagen miRNeasy purification columns (Qiagen, UK) according to manufacturer's specifications. RNA concentration was determined using a NanoDrop™ spectrophotometer. The ratio of absorbance between 260 nm/280 nm for all samples was over 2. RNA was reverse transcribed into cDNA using a RevertAid First Strand cDNA Synthesis Kit (Thermo Fisher, UK) according to manufacturer's specifications. qRT-PCR was performed using the SYBR® Green system (Thermo Fisher, UK) according to manufacturer's instructions in the 7500 Fast Real-Time PCR (Applied Biosystems). mRNA transcript levels of unknown genes were determined by the threshold cycle $\Delta\Delta C_t$ method and normalised to ribosomal protein L19 and glucose-6-phosphade dehydrogenase expression, which were not influenced by exposure to chronic long-term hypoxia. All primer sequences can be found in the Major Resources Table.

Plasma and urine analyses

Plasma concentrations of soluble fms-like tyrosine kinase 1 (sFlt-1), soluble endoglin (sEng), placental growth factor (PlGF), vascular endothelial growth factor (VEGF) and creatinine and urine concentrations of albumin and creatinine were measured using commercially available colorimetric kits, according to manufacturer's instructions. A list of kits used can be found in the Major Resources Table. For sFlt-1, the inter- and intra-assay coefficients of variation were <12.0% and <10.0%, respectively, and the lower limit of detection was 0.1 ng/ml. For sEng, the inter- and intra-assay coefficients of variation were <15.0% for both, and the lower limit of detection was 0.1 ng/ml. For PlGF, the inter- and intra-assay coefficients of variation were <10.0% for both, and the lower limit of detection was 1.0 pg/ml. For VEGF, the inter- and intra-assay coefficients of variation were <15.0% and <10.0%, respectively, and the lower limit of detection was 1.0 pg/ml units. For creatinine, the inter- and intra-assay coefficients of variation were < 5.0% for both, and the lower limit of detection was 2.0 µg/ml. For albumin, the inter- and intra-assay coefficients of variation were <5.0% and <3.0%, respectively, and the lower limit of detection was 2 µg/ml. Measurements from plasma samples taken on 103 and 105 dGA before the onset of chronic normoxia or hypoxia were averaged as baseline measurements.

Longitudinal maternal arterial blood pressure and uterine vascular resistance

A second cohort of pregnant ewes were surgically prepared with catheters and flow probes to permit continuous monitoring of arterial blood pressure and uterine blood flow in N and H groups via the CamDAS wireless data acquisition system [3-5]. In this second cohort, recording during chronic normoxia or chronic hypoxia occurred for 10 days, from 125 to 135 dGA.

Surgery. The second cohort of pregnant Welsh mountain ewes carrying singleton fetuses underwent laparotomy at 116 ± 1 dGA for instrumentation with the wireless data acquisition system under general anesthesia, as previously described [3-5]. Animals were fasted for 24h prior to surgery with ad libitum access to water. On the day of surgery, animals were induced using Alfaxan (1.5-2.5 mg/kg alfaxalone, intravenously; Jurox Ltd., UK) into the jugular vein and then intubated (Portex cuffed endotracheal tube; Smiths Medical International Ltd., UK) using a laryngoscope for maintenance under general anesthesia using 1.5-2.0% isofluorane (IsoFlo; Abbott Laboratories Ltd., UK) in 60:40 oxygen:nitrogen using a positive pressure ventilator (Datex-Ohmeda Ltd., UK). Following induction, the maternal abdomen, flanks and medial surfaces of the hind limbs were shaved and cleaned, and an antibiotic (30 mg/kg procaine benzylpenicillin intramuscularly.; Depocillin; Intervet UK Ltd., UK) and an analgesic (1.4 mg/kg carprofen subcutaneously.; Rimadyl; Pfizer Ltd., UK) were administered. The ewe was then transferred to the surgery theatre and general anesthesia was maintained, as before. The animal was covered with sterile drapes and a midline abdominal incision was made, as described previously [7,8]. A Transonic flow probe (MC2RS-JSF-WC120-CS12-GCP, Transonics, UK) was positioned around the maternal uterine artery, as before, and then exteriorised through a keyhole incision in the ewe's right flank for connection to the wireless data acquisition system [3-5]. Following closure of the abdominal cavity, catheters were inserted via the maternal femoral vein (inner diameter 0.86 mm, outer diameter 1.52 mm; Critchly Electrical Products, Australia) into the maternal inferior vena cava, and via the maternal femoral artery (inner diameter 1.00 mm, outer diameter 1.60 mm; Altec, UK) into the maternal descending aorta. Catheters were exteriorised through a keyhole incision in the maternal left flank and connected to the wireless data acquisition system. While under general anesthesia, the ewe was then fitted with a bespoke jacket housing the wireless data acquisition system. After the end of anaesthesia, the ewe continued to be ventilated until spontaneous respiratory movements were observed, after which the ewe was extubated.

CamDAS^{TM} System. The wireless data acquisition system has been previously described in detail [3,4]. In brief, the CamDASTM (Maastricht Instruments, the Netherlands) consisted of a pressure box attached to one side of the ewe containing pressure transducers (COBE; Argon Division, Maccim Medical, USA) connected to catheters, and a miniaturised flow module on the other side connected to Transonic flow probes (Figure 1B in main text). The pressure and flow boxes were powered by Lithium batteries housed within the same jacket, allowing continuous wireless transmission and recording of maternal uterine blood flow and maternal arterial blood pressure beat-by-beat onto a laptop computer via Bluetooth technology. The weight of the CamDASTM system is less than 2 kg, thereby equivalent to the ewe carrying twins.

Post-surgical recovery. Ewes were allowed to recover in a floor pen adjacent to other sheep with free access to hay and water and a 12:12 h light-dark cycle. Ewes were fed concentrates once a day (200g sheep nuts no. 6; H & C Beart Ltd., UK). Antibiotic (30 mg/kg procaine benzylpenicillin intramuscularly; Depocillin; Intervet UK Ltd., UK) and analgesic (1.4mg/kg carprofen subcutaneously; Rimadyl; Pfizer Ltd., UK) agents were administered to the ewe for five days following surgery. From 120 dGA ewes were fed the daily maintenance diet (5g hay/kg and 40g sheep nuts/kg; Manor Farm

Feeds Ltd., Oakham, UK) and pregnancies were randomly assigned to chronic normoxia (N) or chronic hypoxia (H) groups, as before.

Chronic hypoxia, CamDAS recording and blood sampling. Following 3 days of post-surgical recovery, H ewes were transferred to the hypoxic chambers to acclimatise under normoxic conditions. Five days after surgery, at 125 dGA, H ewes were gradually subjected to hypoxia, reaching 10 ± 1 % inspired oxygen over 24 h, as before. Exposure to chronic hypoxia in this second cohort of surgically prepared H ewes lasted 10 days, until 135 dGA. In both N and H ewes, arterial blood samples were taken daily to measure maternal blood gases, acid-base excess and metabolic status, as previously described[7]. Continuous CamDAS^{TM} recordings of maternal arterial blood pressure and uterine blood flow were converted into minute averages off-line. Uterine vascular resistance was calculated following Ohm's principle by dividing maternal arterial blood pressure by uterine blood flow [5]. Blood gas and acid base values were measured using an ABL5 blood gas analyser (Radiometer; Copenhagen, Denmark; maternal measurements corrected to 38°C). Values for arterial oxygen saturation (Sat Hb) and haemoglobin (Hb) were determined using a haemoximeter (OSM3; Radiometer). Blood glucose and lactate concentrations were measured using an automated analyser (Yellow Springs 2300 Stat Plus Glucose/Lactate Analyser; YSI Ltd., Farnborough, UK). Values for Hct were obtained in duplicate using a microhaematocrit centrifuge (Hawksley, UK). Measurements from blood samples taken between 120 and 124 dGA before the onset of chronic normoxia or hypoxia were averaged as baseline measurements. At the end of *in vivo* experiments, N and H ewes were transported to the post-mortem laboratory and humanely killed as before. The position of the uterine Transonic flow probe and maternal catheter tips was verified.

Statistical analysis

Appropriate power calculations derived from previous data sets were performed to determine the minimum sample size required to achieve statistical significance. Animals exposed to treatment were randomly chosen. Scientists measuring *ex vivo* outcomes were blinded to treatments. All data are expressed as mean ± SEM. The effect of treatment was analysed using the Student's *t* test for unpaired data. The effects of treatment, time and interactions between treatment and time were compared by two-way multiple comparison ANOVA. For all comparisons, values of $P < 0.05$ were accepted as statistically significant. The software used was Graphpad Prism 7 (RRID:SCR_002798).

Supplemental References

1. Kilkenny C, Browne WJ, Cuthill IC, Emerson M, et al. Improving Bioscience Research Reporting: The ARRIVE Guidelines for Reporting Animal Research. PLoS Biology. 2010;8:e1000412. doi: 10.1371/journal.pbio.1000412

2. Brain KL, Allison BJ, Niu Y, Cross CM*, et al.* Induction of controlled hypoxic pregnancy in large mammalian species. *Physiological Reports*. 2015;3:e12614. doi: doi:10.14814/phy2.12614

3. Allison BJ, Brain KL, Niu Y, Kane AD*, et al.* Fetal in vivo continuous cardiovascular function during chronic hypoxia. *The Journal of Physiology*. 2016;594:1247-1264. doi: 10.1113/JP271091

4. Shaw CJ, Allison BJ, Itani N, Botting KJ*, et al.* Altered autonomic control of heart rate variability in the chronically hypoxic fetus. *The Journal of Physiology*. 2018;596:6105-6119. doi: 10.1113/jp275659

5. Jellyman JK, Gardner DS, Fowden AL, Giussani DA. Effects of dexamethasone on the uterine and umbilical vascular beds during basal and hypoxemic conditions in sheep. *American journal of obstetrics and gynecology*. 2004;190:825-835. doi: 10.1016/j.ajog.2003.09.046

Supplementary Tables

	N		H	
	baseline	135 dGA	baseline	135 dGA
PaO_2 (mmHg)	104.2 ± 1.9	98.0 ± 5.0	105.7 ± 3.7	42.0 ± 1.2 *†
$PaCO_2$ (mmHg)	32.3 ±1.3	30.5 ± 2.5	33.6 ± 1.4	32.0± 1.5
Sat Hb (%)	102.6 ± 1.3	101.6 ± 3.5	103.5 ± 0.9	79.5 ± 6.1*†
Haematocrit (%)	28.6 ± 1.1	28.5 ± 0.9	30.0 ± 1.2	33.9 ± 1.0 *†
Haemoglobin (g/dL)	9.41 ± 042	9.20 ± 0.30	10.15 ± 0.08	11.23 ± 0.17 *†
pH	7.49 ± 0.03	7.46 ± 0.06	7.50 ± 0.01	7.48 ± 0.01
Glucose (mmol/L)	2.60 ± 0.14	2.21 ± 0.03	2.78 ± 0.20	2.49 ± 0.22
Lactate (mmol/L)	0.56 ± 0.11	0.51 ± 0.06	0.61 ± 0.11	0.58 ± 0.06

Table S1. *Maternal arterial blood gas, acid base and metabolic status.*
*Values are mean ± SEM for maternal arterial blood gas and, acid base and metabolic status at baseline and at 135 dGA. Groups are N (n=5) and H (n=5). Significant differences (P<0.05) are *N vs. H, or † vs baseline; two-way RM ANOVA.*

Supplementary Figures

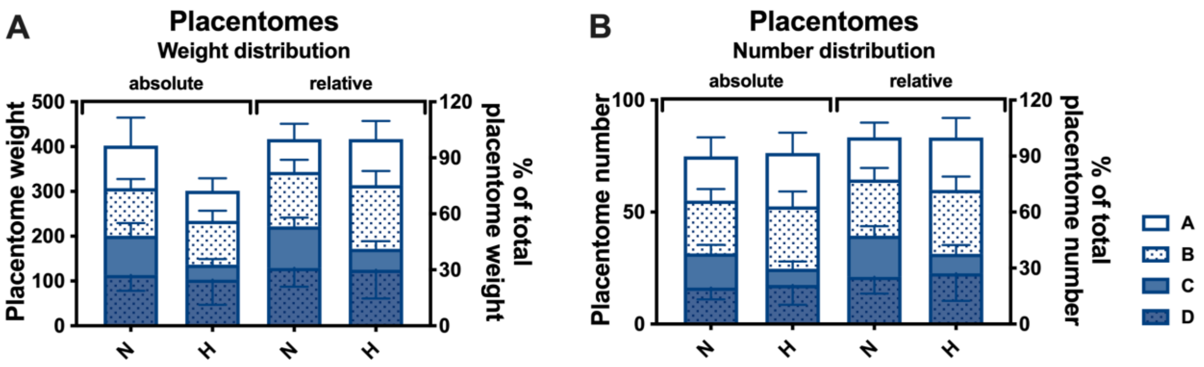

Figure S1. Hypoxic pregnancy does not alter placentome distribution. *Values are mean ± SEM for the absolute and relative weight (A) and number (B) of A-, B-, C- and D-type placentomes. Groups are N (n=8) and H (n=6). There were no significant differences within or between groups.*

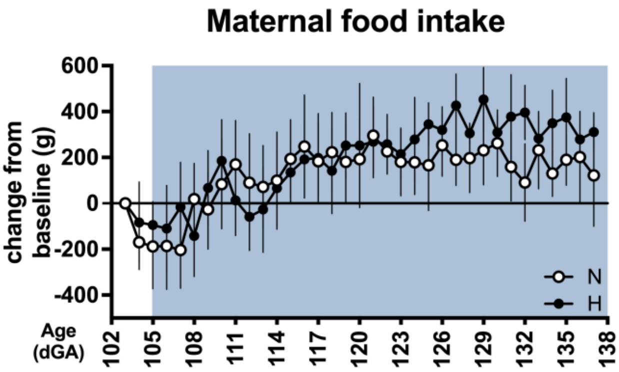

Figure S2. Hypoxic pregnancy does not alter maternal food intake. *Values are mean ± SEM for the change in maternal food intake compared to baseline. Groups are N (○, n=9) and H (●, n=7). There were no significant differences within or between groups.*

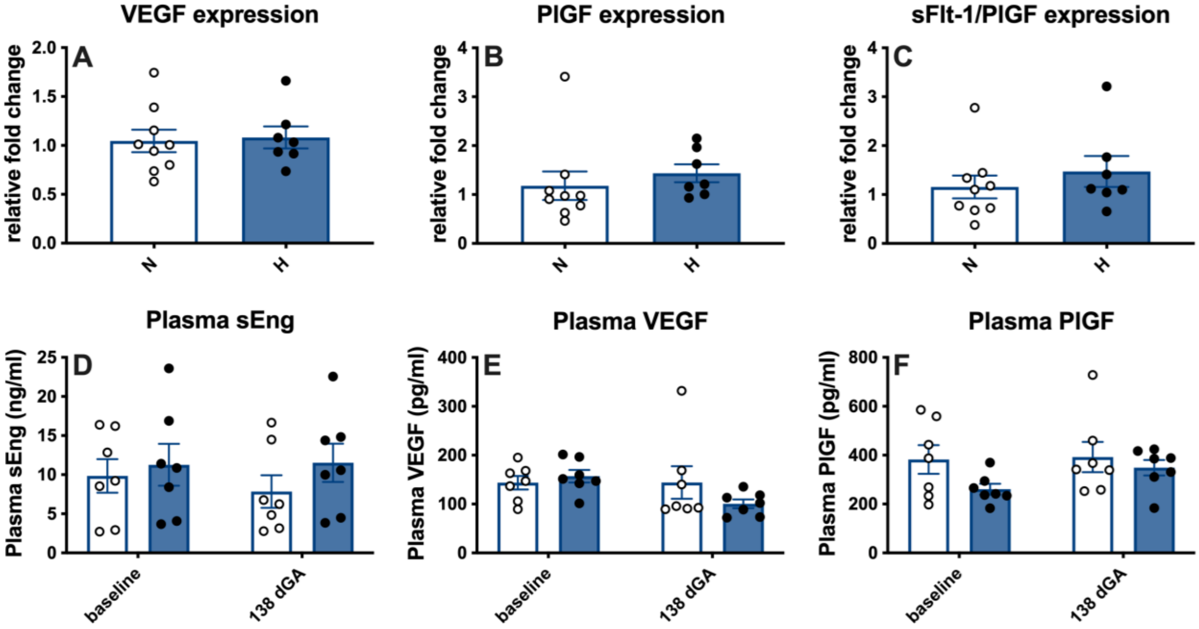

Figure S3. Effects of hypoxic pregnancy on placental gene expression and maternal plasma concentrations of angiogenic factors. *Upper panel: Values are mean ± SEM for the relative fold change for VEGF (A), PlGF (B) and the ratio of sFlt-1 to PlGF (C) in placentomes at 138 dGA as measured by qRT-PCR. Lower panel: Values are mean ± SEM for the maternal plasma concentration of sEng (D), VEGF (E) and PlGF (F) at baseline and at 138 dGA as measured by ELISA. Groups are N (○, n=7-9) and H (●, n=7).*

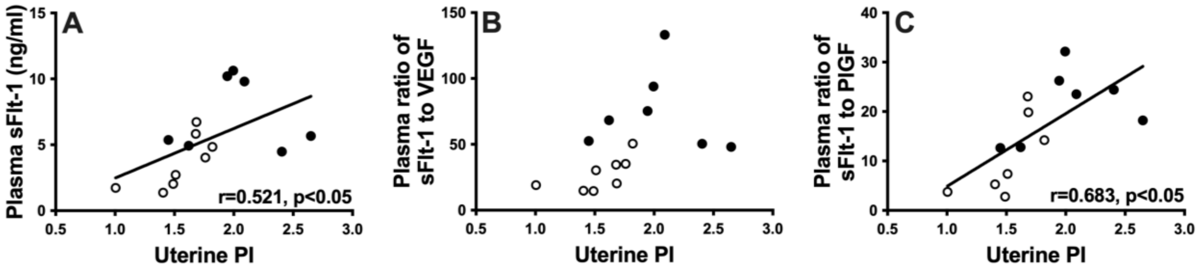

Figure S4. Maternal angiogenic imbalance correlates with uteroplacental vascular resistance. *Correlation of maternal uterine artery PI with the plasma concentration of sFlt-1 (A), with the plasma ratio of sFlt-1 compared to VEGF (B) and with the plasma ratio of sFlt-1 compared to PlGF (C) at 138 dGA. Groups are N (○, n=7-8) and H (●, n=7). Significant correlation (P<0.05) determined by Pearson's correlation.*

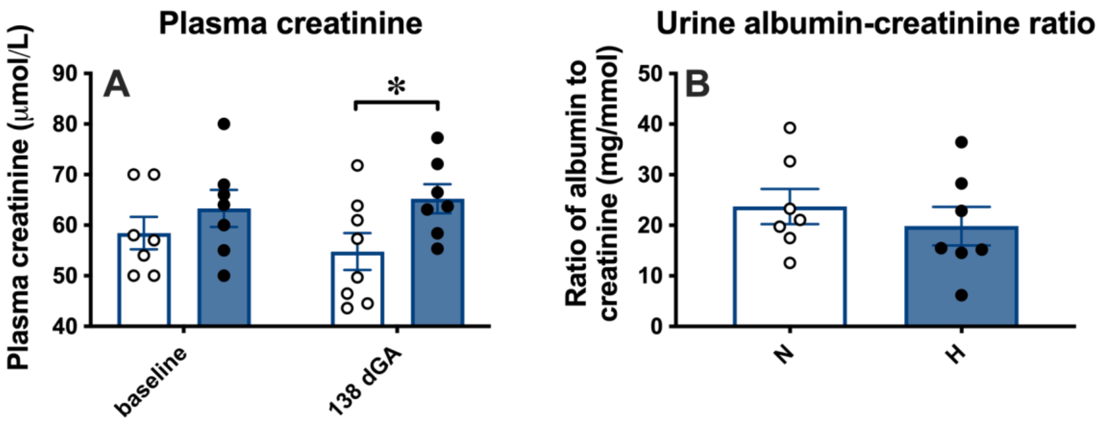

Figure S5. Maternal renal function. *Values are mean ± SEM for the plasma concentration of creatinine during baseline and at 138 dGA (A) and the ratio of albumin to creatinine concentration in maternal urine at 138 dGA (B). Groups are N (○, n=7) and H (●, n=7).*
